# New mechanism to promote long-term T-cell immunity by telomere transfer from antigen-presenting cells

**DOI:** 10.1038/s41423-022-00949-z

**Published:** 2022-11-18

**Authors:** Meiling Jin, Jian-Dong Huang

**Affiliations:** 1 Chinese Academy of Sciences (CAS) Key Laboratory of Quantitative Engineering Biology, Shenzhen Institute of Synthetic Biology, Shenzhen Institutes of Advanced Technology, Chinese Academy of Sciences, Shenzhen, China; 2 School of Biomedical Sciences, Li Ka Shing Faculty of Medicine, The University of Hong Kong, Pokfulam, Hong Kong SAR, China; 3 Department of Clinical Oncology, Shenzhen Key Laboratory for Cancer Metastasis and Personalized Therapy, The University of Hong Kong-Shenzhen Hospital, Shenzhen, China; 4 Guangdong-Hong Kong Joint Laboratory for RNA Medicine, Sun Yat-Sen University, 510120 Guangzhou, China

**Keywords:** Cell biology, Immunology

Telomeres protect the ends of chromosomes and are important biomarkers of cellular aging. The mechanism of telomere elongation in T cells is not clear. A recent study revealed that memory CD4^+^ T cells can acquire telomeric DNA transferred via vesicles from antigen-presenting cells, which inhibits T-cell senescence and promotes T-cell expansion and long-lived immunity.

Maintaining telomere length is important for preventing T cells from undergoing senescence [1]. Upon antigen stimulation, naive T cells differentiate into memory T cells and effector T cells and then undergo telomere shortening, which results in the upregulation of telomerase activity to elongate telomeres [2]. However, telomerase activity is not preserved during sustained antigen stimulation. Consequently, senescent T cells are unable to function, resulting in lower T-cell receptor (TCR) diversity [3, 4]. An alternative mechanism is needed to prevent telomere loss in T cells to prolong their lifespan and delay aging.

In a recent study published in *Nature Cell Biology* by Lanna and colleagues [5], CD4^+^ T cells (naive and central memory cells) were able to elongate their telomeres by acquiring telomeres from vesicles following immunological synapse formation in the presence of an antigen pool (Epstein‒Barr virus, influenza and cytomegalovirus lysates). Telomere elongation was independent of telomerase, which was confirmed by the use of telomerase-deficient T cells. Using DNA labeling and telomere tracking, the researchers found that the release of telomere vesicles and telomeric DNA-containing vesicles from APCs depended on TCR-mediated activation of APCs and calcium flux in donor APCs. Surprisingly, fluorescently labeled telomeric DNA from APC was captured by recipient T cells via vesicle transfer. However, telomere extension in T cells did not require TCR/MHC II activation. These findings suggest a new mechanism involving telomere vesicle transfer from donor APCs to T cells that maintains their telomere length and protects them from senescence.

**Fig. 1:**
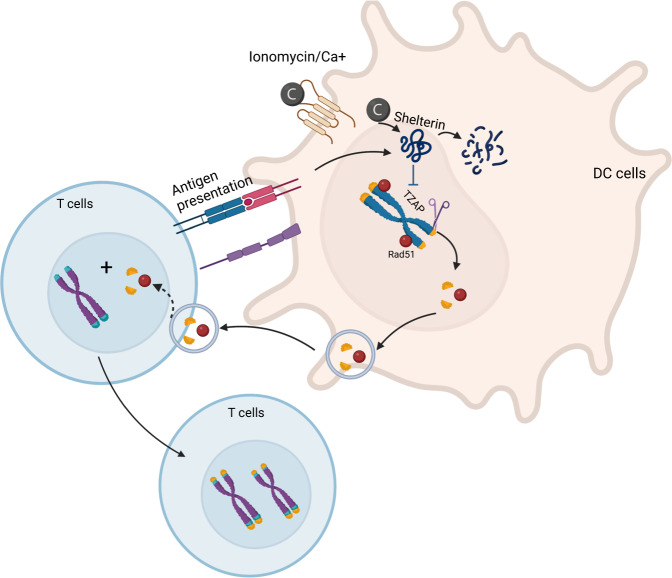
Telomere vesicle formation and transfer to T cells. Activation of APCs by ionomycin or the formation of immune synapses with T cells induces the degradation of shelterin, resulting in increased activation of TZAP and telomeric repeat sequences, which are encapsulated into telomere vesicles together with the protein Rad51. T cells acquire telomere vesicles to lengthen their telomeres, promoting their longevity

To reveal the mechanism by which the APC donor cells transferred telomeres to recipient T cells, the investigators examined the content of the telomere vesicles. The telomere vesicles contained a critical telomere trimming factor, TZAP. The expression and activation of TZAP in APCs were found to be increased upon ionomycin activation or after immune synapse formation. TZAP was necessary for cleavage of telomeric repeat sequences and subsequent encapsulation into vesicles for transfer to T cells (Fig. 1), as vesicle formation and transfer did not occur in TZAP-deficient APCs. In addition, proteasomal degradation of shelterin was shown to be increased in TCR-stimulated APCs, contributing to telomere vesicle release. It has been reported that removal of the telomeric shelterin complex allows TZAP to access and cleave telomeric DNA, after which TZAP localizes to telomeres [6]. The telomere vesicles also contained Rad51, an enzyme required for homologous recombination and telomere elongation. In Rad51-depleted APCs, the count and size of vesicles did not change, but the recombinant function of vesicles was reduced. The Rad51-deficient vesicles showed a reduced ability to elongate T-cell telomeres, resulting in shortened chromosome ends.

Lanna and colleagues showed that telomere transfer also had protective effects against T-cell aging. The researchers found that functional telomere vesicles containing TZAP and Rad51 from activated APCs prevented T cells from undergoing senescence in vitro. T cells with ultrashort telomeres became senescent, whereas those that acquired telomeres from activated APCs. Furthermore, telomere transfer preserved T-cell senescent memory, which was strongly reduced in senescent cells. Moreover, naive T cells were induced to differentiate into stem-like T cells and central memory T cells. However, not all T cells had a high capacity to acquire telomeres from activated APCs.

The researchers investigated the telomere vesicle transfer system in vivo via OT-II ovalbumin antigen-specific challenge in mice. In animals injected with OVA, CD4^+^ T cells acquired telomeres from APCs primed with OVA. The researchers confirmed that Rad51 was required for telomere vesicle transfer and telomere recombination in T cells. After long-term vaccination, CD4^+^ T cells acquiring telomeres expanded more than those that did not acquire telomeres or when Rad51 was depleted. This implies that telomere transfer promotes strengthening of immunological memory. To examine the immune defense potential of these memory cells, mice were challenged with FLUAD influenza vaccination, and the CD4^+^ T-cell memory response was measured. Telomere transfer to T cells protected mice from long-term influenza challenge rather than acute influenza infection, confirming that telomere transfer in T cells induces chromosome extension to provide long-lasting immune protection.

In conclusion, telomere shortening is a feature of senescent T cells that leads to defects in proliferation and effector functions. The findings from this work provide a new mechanism of T-cell telomere elongation involving telomere transfer from APCs, which can prevent T-cell senescence without telomerase. However, it is a mystery why only some rare types of T cells have a high capacity to acquire telomere vesicles. Further studies are needed to reveal the mechanisms of telomere release and vesicle transfer to T cells. Moreover, future research should focus on whether the control and regulation of functional telomere release and acquisition can rescue additional types of immune cells from aging.
